# Left Ventricular Non-Compaction Syndrome Misdiagnosed as Dilated Cardiomyopathy on Several Occasions, Presenting With Recurrent Stroke

**DOI:** 10.14740/cr323w

**Published:** 2014-02-27

**Authors:** Rohan Mandaliya, Margot Boigon, Nneka Nweke, Jeffrey Fierstein

**Affiliations:** a Department of Internal Medicine, Abington Memorial Hospital, Abington, PA 19001, USA; b Division of Cardiology, Abington Memorial Hospital, Abington, PA 19001, USA

**Keywords:** Left ventricular non-compaction, Cardiomyopathy, Echocardiography, Contrast echocardiography

## Abstract

A 57-year-old African American female with a history of ischemic cardiomyopathy and a recent stroke with no residual deficits presented with apraxia and confusion. Non-contrast CT scan of the head revealed multiple embolic strokes in both cerebral hemispheres. Transthoracic echocardiography raised the suspicion for increased trabecular meshwork in the left ventricle. Cardiac MRI confirmed the findings of isolated left ventricular non-compaction (LVNC) syndrome. A contrast-enhanced transesophageal echocardiogram demonstrated the characteristic features of this unusual disease with the additional demonstration of contrast filling the trabecular meshwork. Interestingly multiple transthoracic echocardiograms in the past had failed to identify myocardial non-compaction. The patient was started on warfarin for prophylactic anticoagulation and an implantable defibrillator was placed to lower the risk of sudden death. LVNC is a rare type of genetic cardiomyopathy characterized by excessively prominent trabeculations and deep inter-trabecular recesses in the ventricle wall. Non-compaction remains frequently overlooked even by experienced echocardiographers. Failure to diagnosis may lead to insufficient treatment since it is often associated with a risk of thromboembolism, life-threatening arrhythmias and sudden death. Furthermore, because of the familial association described with ventricular non-compaction, screening of first relatives with echocardiography is recommended.

## Introduction

Left ventricular non-compaction (LVNC) is a rare type of genetic cardiomyopathy. It has been associated with significant risk of developing arrhythmias, thromboembolic complications, severe systolic dysfunction and sudden cardiac death [1]. Echocardiography plays an important role in diagnosing the condition based on the proposed criteria [2]. However, the diagnosis can be overlooked depending on the image quality as well as the skills and experience of the investigator. In a recent case series, diagnosis was initially missed in almost 90% of the cases [3]. Recently, cardiac MRI has been studied and utilized to better visualize and confirm the findings of non-compaction syndrome. The purpose of this case report is: 1) to review a single case demonstrating this rare cardiomyopathy, 2) to highlight the significance of truly diagnosing this condition as it is associated with severe complications, 3) to highlight the role of multimodality diagnostic approach including contrast echocardiography in correct diagnosis and 4) to review the literature regarding the genetics and management of this condition.

## Case Report

A 57-year-old female was brought to the hospital because of new onset of confusion and apraxia. The patient had a past medical history significant for nonischemic cardiomyopathy for 2 years. The patient had a stroke several weeks prior during an admission to another facility. At that time, work-up for the etiology of the stroke was negative except for cardiomyopathy with an ejection fraction of 30%. The patient did not have any residual focal motor neurologic deficits, although she was cognitively impaired. Medications at discharge included aspirin, carvedilol, lisinopril and atorvastatin.

On admission, the patient was hemodynamically stable without any distress. Her speech was incoherent and tangential. She had difficulty identifying objects and could not use eating utensils appropriately. Pupils were equal and reactive without any nystagmus or gaze disturbance. No other neurologic deficits were present. On cardiac auscultation, first and second heart sounds were heard normally without any murmur. Lungs were clear. Carotid and other peripheral pulses were palpable and symmetric. Trace bilateral ankle edema was present.

**Figure 1:**
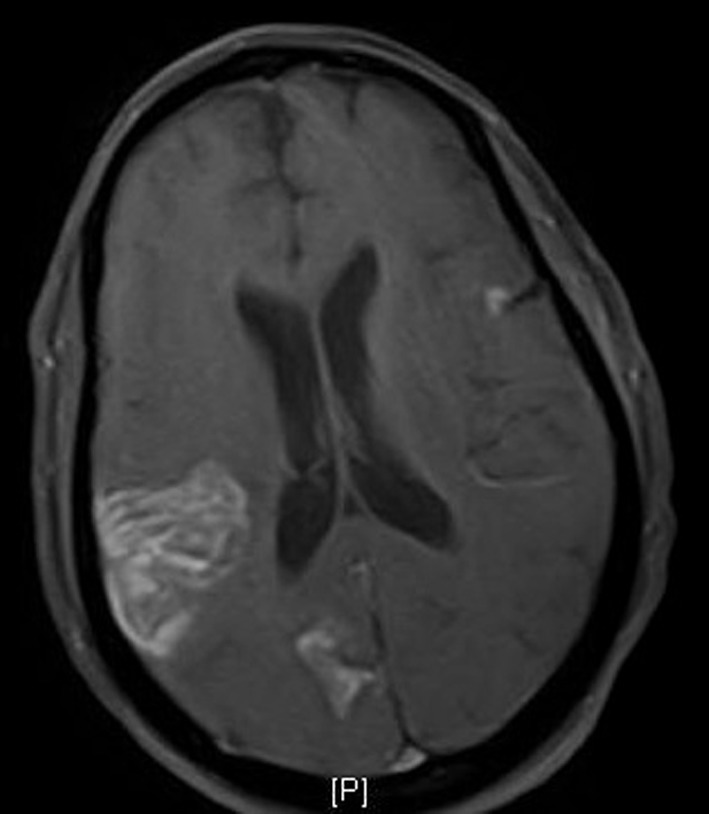
MRI of the brain shows acute and subacute infarcts in the frontal, parietal and occipital lobes.

The patient had a normal complete blood count, electrolytes, liver function and thyroid function tests. The cardiac BNP value was 340 pg/mL. Electrocardiogram revealed normal sinus rhythm with T wave inversions in the lateral leads. A chest X-ray was negative for active cardiopulmonary disease. Non-contrast CT scan of the head showed areas of low attenuation in the right posterior frontal and parietal regions consistent with subacute infarctions which were outlined with high attenuation representing hemorrhagic conversion. Subsequent MRI was consistent with these findings with additional tiny acute infarcts in the parietal lobes (Fig. 1).

**Figure 2:**
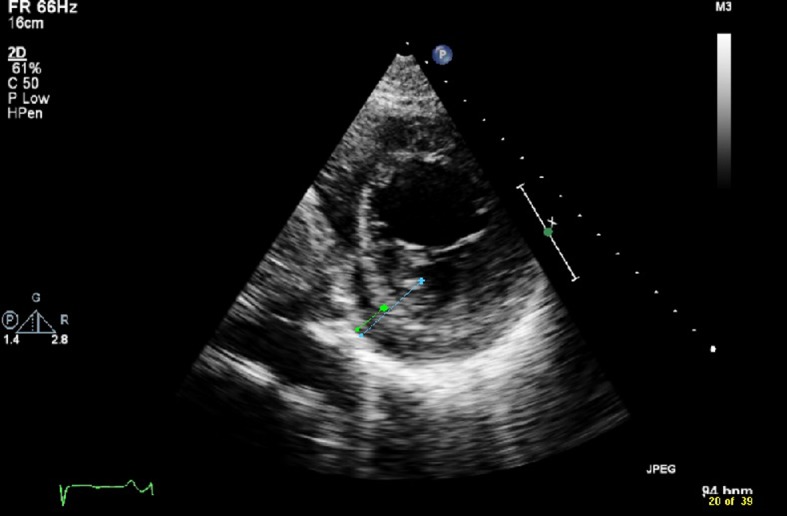
Transthoracic echocardiogram with short parasternal view shows prominent trabecular projections with the distance from the epicardial surface to the trough of the trabecular recess X (green line) of 1.3 cm, and with the distance from the epicardial surface to the peak of trabeculation Y (blue line) of 3 cm with ratio X/Y < 0.5.

**Figure 3:**
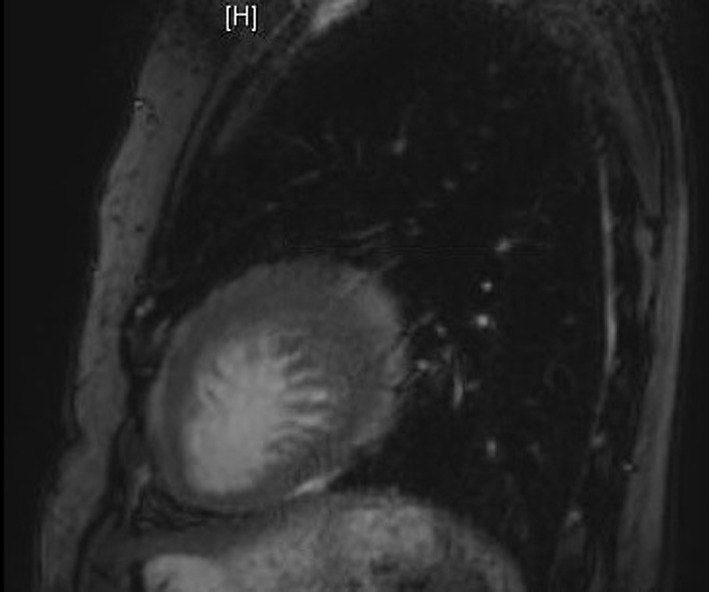
Cardiac MRI shows prominent trabecular processes in the left ventricular cavity.

**Figure 4:**
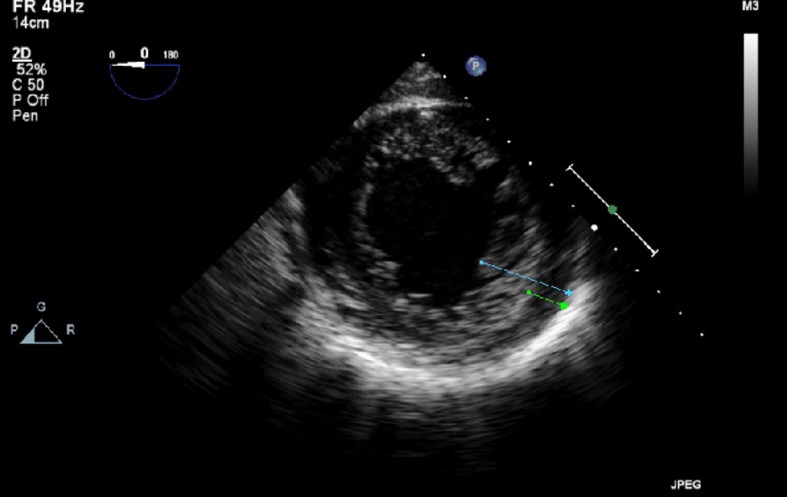
Transesophageal echocardiogram with trans gastric view without contrast shows prominent trabecular projections with the distance from the epicardial surface to the trough of the trabecular recess X (green line) of 0.85 cm, and with the distance from the epicardial surface to the peak of trabeculation Y (blue line) of 2.6 cm with ratio X/Y < 0.5.

**Figure 5:**
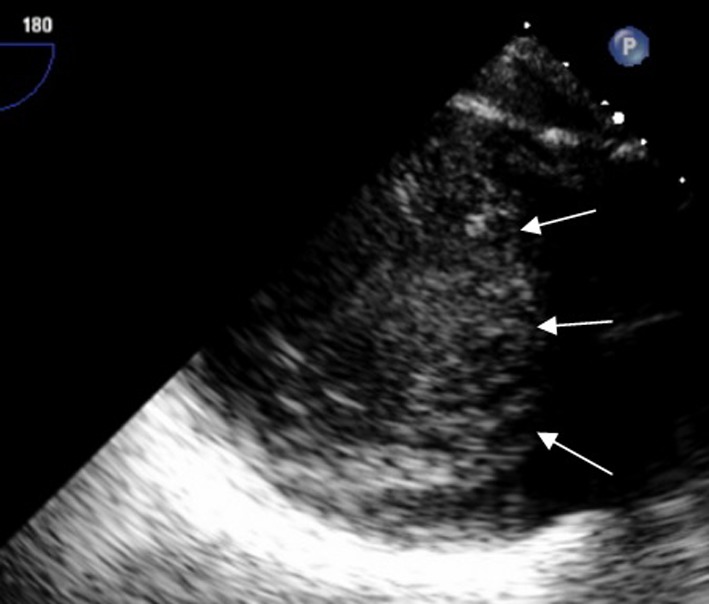
Transesophageal echocardiogram with trans gastric view with contrast demonstrates the contrast filling in the inter-trabecular recesses within the ventricular cavity.

A transthoracic echocardiogram for an embolic source showed severe reduction of the left ventricular systolic function with ejection fraction of 10-15%. On close observation, there was a suspicious appearance of prominent trabeculations in the left ventricle (Fig. 2). Cardiac MRI showed signs of non-compaction cardiomyopathy with prominent trabeculations in the left ventricular myocardium (Fig. 3). A transesophageal echocardiogram with contrast showed more clearly the prominent trabeculations with contrast occupying recesses between the trabeculae (Fig. 4, 5). A diagnosis of isolated LVNC syndrome was established which caused recurrent embolic stroke in the patient. Continuous telemetry revealed episodes of nonsustained ventricular tachycardia. An implantable cardiac defibrillator (ICD) was placed. The patient was started on systemic anticoagulation with warfarin to lower the risk of systemic embolism and recurrent stroke. Since non-compaction has a familial occurrence, the patient’s two sisters were recommended to undergo screening echocardiography. The patient was discharged home with resolution of her neurologic deficits.

## Discussion

Isolated LVNC is an uncommon finding and the true prevalence of it remains unknown. It was first described by Chin et al in 1990 [1]. It is characterized by persistent embryonic myocardial morphology with prominent trabeculations and deep inter-trabecular recesses that extend into the ventricular wall. There is no communication with the coronary circulation and the abnormalities exist in the absence of other cardiac anomalies which would explain the abnormal development [1, 4]. LVNC is hypothesized to result from premature cessation of the compaction of the trabecular meshwork during the embryologic phase. During early embryonic development, the myocardium is a loose network of interwoven fibers separated by deep recesses that link the myocardium with the left ventricular cavity. Gradual “compaction” of this spongy meshwork of fibers and inter-trabecular recesses, or “sinusoids”, occurs between week 5 and week 8 of embryonic life, proceeding from the epicardium to endocardium and from the base of the heart to the apex [1, 4-6]. The coronary circulation develops concurrently during this process, and the inter-trabecular recesses are reduced to capillaries.

To date, the arrest in the embryonic endomyocardial morphogenesis is believed to be the mechanism underlying the cardiomyopathy. This is supported by genetic factors as well as from murine data, in which mice with mutations of these genes developed LVNC [7, 8]. This hypothesis, however, has been challenged by the detection of a normal phenotype in infancy that was subsequently diagnosed with LVNC [9]. At the same time, it is unclear whether these findings were related to limitations of fetal echocardiography or development of the syndrome at a later phase.

LVNC is a genetically heterogenous disorder, with both X linked as well as autosomal dominant inheritance described. Because of the possible genetic influence, there has been emphasis on the screening of all first-degree family members of the affected individuals. Mutations in the G4.5 gene that encodes the tazaffin family of proteins result in infantile X linked cardiomyopathies, including Barth syndrome with dilated cardiomyopathy and LVNC in a pediatric patients [9-11]. These mutations are not found in the adult population. Mutations have been described in adult LVNC in the genes encoding a-dystrobrevin and ZASP, intergral parts of the complex which links the extracellular matrix of the myocardial cell to the cytoskeleton [11, 12]. Sarcomere gene mutations have been found in common with in patients with hypertrophic, dilated and isolated LVNC. A novel missense mutatin, Pe96k mutation, in the cardiac troponin gene has been described in a family with LVNC [13]. There is ongoing research to understand the genetics of the non-compaction syndrome.

The four major clinical manifestations of non-compaction that have been described to date include heart failure, arrhythmias, embolic events and sudden death [1, 5, 14]. While asymptomatic patients are diagnosed incidentally or during family screening, most of the symptomatic patients present mainly as a result of systolic/diastolic ventricular dysfunction which includes nonspecific chest pain/discomfort, heart failure symptoms or arrhythmias.

Arrhythmias that are associated with LVNC include ventricular tachyarrhythmias (47%), atrial fibrillation (25%), paroxysmal supraventricular tachycardia, complete heart block and sudden cardiac death (accounted for more than 50% of deaths) [1, 2, 4, 14]. Ventricular arrhythmias and sudden cardiac death are serious manifestations of LVNC. The incidence of sustained ventricular tachycardia has been from 0% to 9%, while the incidence of nonsustained ventricular tachycardia ranged from 20 to 33% over 2.3 - 3.8 years in published studies [15]. Sudden cardiac death is responsible for the majority of the deaths in patients with non-compaction [14]. Li et al recently described two cases of young adults who died suddenly. Their post mortem examination revealed that both the patients died of undiagnosed LVNC [16].

Systemic embolic events including stroke as seen in our patient can be associated with LVNC because deep inter-trabecular recesses with slow and sluggish blood flow with stasis increase the risk of thrombus formation.

### Diagnosis

The diagnosis of LVNC is primarily based on 2D echocardiographic findings. Chin et al first proposed diagnostic criteria for non-compaction syndrome in 1990, followed then by Jenni and Stollberger [1, 17, 18]. Table 1 shows proposed diagnostic criteria by the respective authors. However, it is interesting to note that there is no universally accepted definition of LVNC.

**Table 1:** Proposed Diagnostic Criteria by Three Different Authors

Chin et al [1]	1) LVNC is defined by a ratio of X/Y < 0.5. 2) X is the distance from the epicardial surface to the trough of the trabecular recess, and Y is the distance from the epicardial surface to the peak of the trabeculation. 3) These focus on trabeculae at the left ventricular apex on the parasternal short-axis and apical views, and on left ventricular free-wall thickness at end diastole.
Jenni et al [17]	1) Absence of coexisting cardiac structural abnormalities. 2) Recesses supplied by intra-ventricular blood on color Doppler. 3) A thickened left ventricular wall consisting of two layers: a thin compacted epicardial layer; and a markedly thickened endocardial layer with numerous prominent trabeculations and deep recesses with a maximum ratio of non-compacted to compacted myocardium > 2:1 at end-systole in the parasternal short-axis view.
Stollberger et al [18]	1) Four or more trabeculations protruding from the left ventricular wall, located apical to the papillary muscles and visible in one imaging plane. 2) Trabeculations with the same echogenicity as the myocardium and synchronous movement with ventricular contractions. 3) Perfusion of the inter-trabecular recesses from the left ventricular cavity. 4) Acquisition of images in the apical four chamber view, atypical views to obtain the best quality image to differentiate between false chords, aberrant bands and trabeculations.

Kohli et al studied all three criteria for identification of LVNC in HF and control groups [19]. There was a poor correlation between the three echocardiographic criteria with only 30% of patients satisfying all of them. Note that 8% of controls met at least one of the diagnostic criteria. This raises the concern that the current echocardiographic criteria are too sensitive and result in overdiagnosis of LVNC. At the same time perhaps due to poor image quality and operator experience, the detection rate may be falsely low and the true diagnosis can be missed as in our case.

Cardiac MRI has been recently utilized as a sensitive tool for the diagnosis of LVNC. It provides a good correlation with transthoracic echocardiography for the localization and extent of non-compaction and is useful in cases with poor echocardiographic image quality. Petersen et al proposed that NC/C > 2.3 in diastole accurately diagnosed LVNC [20].

Recently contrast echocardiography has been utilized as an extension of the existing routine echocardiographic examination. It is a low-cost, real-time, non-invasive technique which shows high correlation in the diagnosis of LVNC. The added advantage of contrast echocardiography is that it gives a real-time assessment of intracardiac blood flow and depicts contrast filling in the inter-trabecular spaces.

We performed a transesophageal echocardiography with contrast to aid in the diagnosis of LVNC. Although transesophageal echocardiography has not been used often for the diagnosis of LVNC, it can be extremely helpful in the diagnosis when transthoracic echocardiography image quality is poor and there is a high suspicion. We suggest that the simultaneous use of contrast echo either transthoracic or transesophageal along with cardiac MRI helps to prove concordance among the findings and highly increases the sensitivity of true diagnosis.

There are no proposed guidelines for the management of patients with LVNC. It seems reasonable that for patients who are asymptomatic without any LV dysfunction, follow-up should be echocardiography and Holter monitoring every 2 or 3 years [21, 22]. Patients who have evidence of LV dysfunction on echocardiography need to be treated with standard medical therapy for heart failure and followed every 1 - 2 years. There is a controversy regarding the prophylactic use of oral anticoagulants in patients with LVNC to prevent embolic complications. Initially Oechslin et al in JACC (2000), proposed to anticoagulate all patients with LVNCM [14]. Based on recent retrospective analysis, anticoagulation has been recommended in patients of LVNC with severe systolic dysfunction ejection fraction < 40%, concomitant atrial fibrillation, previous history of embolic events, or presence of ventricular thrombi [21-23].

The concern for ICD implantation in all LVNC patients has been proposed due to high risk of sudden cardiac death. There are no clear cut guidelines for ICD implantation for patients with LVNC. In the present era of knowledge an ICD should be implanted in patients with LVNC presenting with symptomatic ventricular arrhythmias, syncope or severely impaired LV systolic function (left ventricular ejection fraction < 35%) [23].

Finally since LVNC is a genetic cardiomyopathy, family screening with echocardiography is recommended for all first-degree relatives. Hoedemaekers et al in their recent study investigated relatives of 50 unrelated LVNC probands and found familial cardiomyopathy in 64% of families screened [24].

### Conclusions

Isolated LVNC cardiomyopathy is a rare form of cardiomyopathy, with many unanswered questions and overall knowledge limited to case reports and case series. It should not be overlooked in patients presenting with nonischemic cardiomyopathy. A multimodality imaging approach may improve the diagnostic accuracy. Contrast echocardiography can significantly improve the yield and detection rates. Functional diagnostic criteria need to be defined apart from the morphologic criteria to better diagnose and determine appropriate management therapy.
